# Extreme Hyperhemolysis Syndrome in a Patient With Sickle Cell Disease Successfully Managed With Eculizumab: A Case of Survival at 1.2 g/dL Hemoglobin

**DOI:** 10.7759/cureus.106034

**Published:** 2026-03-28

**Authors:** Isa Cordeiro, Carolina Cerca, Marta Pinheiro, Claudia Pratas, Nuno Germano

**Affiliations:** 1 Intensive Care Unit, Unidade Local de Saúde de São José, Lisbon, PRT

**Keywords:** eculizumab, hyperhemolysis syndrome, oxygen consumption, severe anemia, sickle cell disease complications

## Abstract

This case presents a 26-year-old male with sickle cell disease (SCD) who experienced a catastrophic hyperhemolysis syndrome (HHS) triggered by red blood cell (RBC) transfusion. The patient was initially hospitalized for a vaso-occlusive crisis and fever; the patient’s clinical status deteriorated rapidly as his hemoglobin (Hb) plummeted from 7.5 g/dL to a nadir of 1.2 g/dL. Despite treatment with corticosteroid and intravenous immunoglobulin (IVIG) therapy, the hemolytic process persisted. To manage the critical imbalance between oxygen delivery and demand, invasive mechanical ventilation was initiated as a supportive metabolic measure. Due to the failure of conventional therapies, eculizumab was administered on the third day of intensive care unit (ICU) admission. This intervention resulted in the cessation of hyperhemolysis, followed by gradual hematological stabilization and recovery. This report underscores the severity of HHS and demonstrates the successful use of terminal complement inhibition as a life-saving rescue strategy in extreme cases.

## Introduction

Hyperhemolysis syndrome (HHS) is a serious and poorly understood complication of blood transfusion in sickle cell disease (SCD). While SCD is characterized by chronic hemolytic anemia and recurrent vaso-occlusive events, the management of its acute complications often necessitates red blood cell (RBC) transfusions. However, in a subset of patients, this intervention triggers a paradoxical reaction in which the post-transfusion hemoglobin (Hb) level falls below the pre-transfusion baseline, occurring in approximately 3% to 5% of those transfused. Despite its severity, HHS remains frequently under-recognized in routine clinical practice, often misdiagnosed as a simple transfusion reaction or a worsening pain crisis. This phenomenon is primarily driven by "bystander hemolysis," an intense immune-mediated process in which hyperactivation of the complement system leads to the destruction not only of the donor’s RBCs but also of the patient’s autologous cells. The pathophysiology of bystander hemolysis remains a challenge for clinicians, as it involves the marking of "innocent" host erythrocytes for destruction, often in the absence of detectable new alloantibodies. Further transfusions often exacerbate hemolysis, making early recognition essential to prevent fatal outcomes. Standard therapeutic approaches, including high-dose corticosteroids and intravenous immunoglobulin (IVIG), often fail to arrest the rapid decline in Hb in refractory cases. This has led to the emergence of salvage therapies aimed at the terminal complement pathway. Eculizumab, a humanized monoclonal antibody, is a potent C5 inhibitor that blocks the cleavage of C5 into its pro-inflammatory and lytic components, preventing the formation of the membrane attack complex (MAC) on the erythrocyte surface. Despite its potential, there is limited evidence on survival at extreme Hb nadirs or on the optimal timing of salvage therapies such as eculizumab. This report describes a survival at an Hb nadir of 1.2 g/dL, managed with terminal complement blockade [1-3].

## Case presentation

A 26-year-old male with SCD, frequent vaso-occlusive crises, and functional asplenia presented to the Emergency Department with bilateral leg pain and fever. His baseline Hb was 7.5 g/dL, and on admission, it was 7.1 g/dL. He reported severe fatigue, tachycardia, and dyspnea with minimal exertion. Due to symptomatic anemia and possible infection, one unit of RBCs was transfused according to hospital protocol for symptomatic patients. Empirical antibiotics were initiated for a suspected respiratory infection.

Over the next 48 hours, despite receiving two additional RBC units, his Hb declined progressively (Day 0: 7.1 g/dL; Day 2: 5.6 g/dL; Day 6: 2.4 g/dL). By day 6 of hospitalization, he was admitted to the Intensive Care Unit (ICU) with extreme fatigue and an Hb of 2.4 g/dL. Laboratory markers confirmed severe intravascular hemolysis: haptoglobin < 0.10 g/L, total bilirubin 2.34 mg/dL (direct 1.22 mg/dL), and lactate dehydrogenase (LDH) 1170 U/L. Notably, the reticulocyte count was 20.2% (immature reticulocyte fraction 33.7%), showing a vigorous but ineffective bone marrow response. Despite the profound drop in Hb, the antibody screening and direct antiglobulin test were negative, a finding consistent with the bystander hemolysis mechanism of HHS, where autologous cell destruction occurs independently of new alloantibody formation.

In the ICU, he was started on IVIG (1 g/kg) and pulse methylprednisolone. Over a 48-hour period, his Hb dropped to a critical 1.2 g/dL as lactate levels climbed to 7 mmol/L, indicating severe tissue hypoxia. Due to the high risk of anoxia, he was intubated and mechanically ventilated to reduce systemic oxygen demand. The team defined "refractory" status as a fall in Hb of more than 1 g/dL per 24 hours despite at least 48 hours of IVIG and steroids, with no alternative causes of hemolysis and persistently elevated LDH. Given the ongoing decrease in Hb and persistent hemolysis, eculizumab (900 mg) was administered on ICU day 3. After the first dose, hemolysis slowed, and Hb stabilized, avoiding further high-risk transfusions. The evolution of Hb, lactate, and LDH levels is summarized in Table 1. As detailed in Table 1, the patient’s clinical deterioration was mirrored by a rapid divergence in biochemical markers. On D3 ICU, the Hb reached a critical nadir of 1.2 g/dL, coinciding with a peak in LDH and a significant lactate spike, reflecting severe tissue hypoxia. Following the initiation of terminal complement blockade on D3, a consistent downward trend in hemolytic markers was observed, with LDH levels halving by D6 and lactate normalizing. The patient showed sustained clinical improvement and was discharged after a 26-day hospital stay with an Hb of 8.0 g/dL.

**Table 1:** Evolution of hematological and metabolic parameters during intensive care unit stay D0: intensive care unit admission

Parameters	Reference values	D0 ICU	D3 ICU	D6 ICU
Hemoglobin g/dL	13.5-17.5 g/dL	2.4	1.2	3.4
Lactate mmol/L	0.5-2.0 mmol/L	0.8	7.0	0.8
Lactate dehydrogenase U/L	140-280 U/L	1170	2152	1800

## Discussion

This case illustrates the extreme severity of HHS, a life-threatening complication where Hb reached 1.2 g/dL. The hallmark of HHS is the "bystander" effect, where the immune system destroys both autologous and donor RBCs. Hyperactivation of the alternative complement pathway appears to drive this process. In SCD, autologous RBCs are more susceptible to complement-mediated lysis due to reduced expression of membrane-bound regulatory proteins such as CD55 and CD59. This deficiency leads to non-specific deposition of the MAC on these cells, resulting in the catastrophic drop in Hb observed in this patient [4,5].

An important feature of this case was persistent, vigorous reticulocytosis (20.2%) despite a sharp drop in Hb. While HHS is commonly associated with transient marrow suppression and reticulocytopenia, this patient maintained a robust regenerative response. This paradox can mislead clinicians, as robust reticulocytosis during ongoing hemolytic anemia may seem reassuring. In HHS, however, it indicates that peripheral destruction exceeds erythropoietic capacity. This confirms that the pathology was due to massive peripheral intravascular destruction rather than central production failure. Aplastic crisis was ruled out based on biochemical markers of massive hemolysis, including a disproportionate rise in LDH and indirect bilirubin, which are not characteristic of primary bone marrow failure. Also, a delayed hemolytic transfusion reaction was excluded as the post-transfusion Hb fell significantly below the pre-transfusion baseline, indicating the destruction of autologous RBCs rather than just the clearance of transfused units [6,7].

Compared to other published HHS cases, most report reticulocytopenia or an inappropriately low reticulocyte count for the level of anemia, which is believed to reflect marrow suppression from immune-mediated attack or transfusion-related factors. However, there are a few documented cases in which severe peripheral hemolysis was paired with unexpectedly strong reticulocytosis, indicating preserved marrow function. Our case adds to this less commonly reported phenotype and suggests that a high reticulocyte count in HHS should not provide false reassurance, as it does not mitigate the risk of life-threatening anemia. This observation points to the need to recognize that reticulocyte response in HHS can be variable, and management decisions should rely on the overall clinical and laboratory picture rather than reticulocyte count alone [8-11].

IVIG and steroids are first-line treatments but often do not prevent rapid activation of the terminal complement pathway. In this setting, eculizumab can be an effective rescue therapy. By binding to C5, eculizumab inhibits MAC formation, likely the main cause of this patient's refractory hemolysis. After administration on ICU day 3, hemolysis slowed, and clinical recovery followed [12,13].

Furthermore, the decision to initiate invasive mechanical ventilation at the Hb nadir was a crucial supportive maneuver to "bridge" the patient until definitive therapy could take effect. In the face of catastrophic oxygen delivery (DO2) failure, mechanical ventilation served as a metabolic bridge. To specifically reduce systemic oxygen demand (VO2), a ventilation strategy was adopted that promoted deep sedation and controlled ventilation, consequently minimizing the work of breathing. This approach, intended to minimize all unnecessary oxygen consumption, was essential to prevent irreversible anoxic organ damage, particularly to the myocardium and central nervous system [14,15].

## Conclusions

HHS is a highly feared and life-threatening complication of blood transfusion in SCD. As shown in this case, Hb nadirs can reach extreme levels (1.2 g/dL), requiring rapid immunomodulation and aggressive supportive care, including mechanical ventilation to reduce oxygen consumption. When IVIG and steroids fail to stop complement-mediated RBC destruction, eculizumab can be a promising salvage therapy to block terminal complement activation. However, the successful outcome in such critical scenarios likely reflects a multimodal approach, including aggressive supportive care and mechanical ventilation to minimize systemic oxygen consumption. While the rapid stabilization observed after eculizumab administration is encouraging, the lack of comparative studies and the nature of case reports limit definitive conclusions regarding its isolated efficacy. Early recognition and avoidance of further transfusions are important to prevent fatal hemolysis. In this case, several critical red flags served as diagnostic anchors. The most definitive sign was the paradoxical drop in Hb below the pre-transfusion baseline. Furthermore, the presence of vigorous reticulocytosis and a negative immunohematological workup strongly suggests a complement-mediated mechanism. 
